# Excess Mortality Related to Chikungunya Epidemics in the Context of Co-circulation of Other Arboviruses in Brazil

**DOI:** 10.1371/currents.outbreaks.14608e586cd321d8d5088652d7a0d884

**Published:** 2017-11-13

**Authors:** André Ricardo Ribas Freitas, Luciano Cavalcanti, Andrea PB Von Zuben, Maria Rita Donalisio

**Affiliations:** Secretary of Health of Campinas, Department of Health Surveillance, Campinas, SP, Brazil; Department of Epidemiology, São Leopoldo Mandic Medical School, Campinas, SP, Brazil; Department of Community Health, Federal University of Ceara, Fortaleza-CE, Brazil; Secretary of Health of Campinas, Department of Health Surveillance, Campinas, SP, Brazil; Department of Public Health, Faculty of Medical Sciences Unicamp, Campinas, SP, Brazil

## Abstract

**Introduction:**

Chikungunya is an emerging arbovirus that reached the Western Hemisphere at the end of 2013. Studies in the Indian Ocean and India suggest that passive surveillance systems cannot recognize many of deaths associated with chikungunya, which can be inferred by an increase in the overall mortality observed during chikungunya epidemics.

**Objective:**

We assess the mortality associated with chikungunya epidemics in the most affected states in Brazil, from 2015 and 2016.

**Methods:**

We studied the monthly mortality by age group, comparing a period without epidemics to a chikungunya epidemic period, which we defined arbitrarily as consecutive months with incidences of more than 50 cases/100,000 persons.

**Results:**

We obtained official data from the National System of Reported Diseases (SINAN) and the Mortality Information System (SIM), both maintained by the Ministry of Health. We identified a significant increase in the all-cause mortality rate during chikungunya epidemics, while there was no similar mortality in the previous years, even during dengue epidemics. We estimated an excess of 4,505 deaths in Pernambuco during the chikungunya epidemics (47.9 per 100,000 persons).The most affected age groups were the elderly and those under 1 year of age, and the same pattern occurred in all the states.

**Discussion:**

Further studies at other sites are needed to confirm the association between increased mortality and chikungunya epidemics indifferent age groups. If these findings are confirmed, it will be necessary to revise the guidelines to recognize the actual mortality associated with chikungunya and to improve therapeutic approaches and protective measures in the most vulnerable groups.

## Introduction

Chikungunya is an emerging arbovirus that reached the Western Hemisphere at the end of 2013. On December 9, 2013, the Pan American Health Organization (PAHO) has issued an alert about the transmission of chikungunya in the Americas^1^. Since then, the transmission of CHIKV were confirmed in 44 countries and territories in the region, with more than 2 million reported cases and 403 deaths. In Brazil, the transmission was confirmed in September 2014, but the worst epidemic occurred in 2016 with 216,102 cases reported in 696 municipalities and 91 deaths.

Studies in Indic Ocean and India suggest that passive surveillance system cannot recognize many of deaths associated with chikungunya, which can be inferred by an increase in the overall mortality observed during chikungunya epidemics 3^,^^4^.

The lethality observed in Brazil and in all America, has been much lower than that reported in other countries^1^, perhaps due to difficulty in diagnosis and lack of adequate tools for surveillances^1^. The objective of this study was to assess the mortality associated with chikungunya epidemics in Brazil by comparing the mortality of pre-chikungunya with mortality during chikungunya epidemics^2^^,^^3^.

## Methods

We studied the 3 Brazilian states with higher chikungunya incidence rates (IR), Pernambuco (9,410,772 inhabitants), Rio Grande do Norte (3,474,998 inhabitants), and Bahia (15,498,733 inhabitants)^5^. Bahia is the largest and most populated state, with a heterogeneous incidence of chikungunya, and therefore we analyzed separately each one of 9 administrative regions of Bahia. We retrospectively analyzed anonymous, publicly available data from the Brazilian Ministry of Health.

To evaluate the possible effect of chikungunya on all-cause mortality, we compared the number of deaths expected for the current estimated population with that observed during the epidemic, during 2015 and 2016. Given the lack of previous data of the CHIKV circulation in the studied regions, we arbitrarily considered as epidemic period onset on first of at least two consecutive months with incidence rates higher than 50 cases per 100 thousand population; and for the end of epidemic period we defined as incidence rate lower than 50 per 100 thousand for two consecutive months for all studied regions. We plot the incidence rate of dengue during 2015 and 2016 in all studied regions for comparing.

The expected mortality rates were calculated based on the mean monthly Age Standardized Mortality Rate (ASMR, using the Brazilian population, census 2010) of the same months between 2011 to 2013, years prior to chikungunya transmission. Values conservative 99% of the upper limit of the confidence interval were calculated for each month as a maximum threshold of the monthly-expected deaths for each studied region. We defined excess deaths as the difference between the observed and expected deaths during the epidemics months.

We also compared the expected and observed ASMR for each month during the chikungunya epidemic period and calculated change in mortality rate per 100,000 and percentage change (%) in each region. Similar methodology has been used previously^2^^,^^3^^,^^4^. In most affected areas, we also calculated the mortality rate for epidemic period and 99% of the upper limit of the confidence interval by age group. We used the mean monthly mortality rate by age group of the same periods of the non-epidemic years 2011-2013 as expected mortality rates.

We obtained population data (census and projections) from the official Brazilian Institute of Geography and Statistics (IBGE), and mortality data from the official Mortality Information System. Probable cases of chikungunya, dengue, and zika virus infection (laboratory and clinical epidemiological criteria) were available in the Brazilian Epidemiological Surveillance System (SINAN), based on cases reported by healthcare professionals. For the processing of data and figures and the statistical analysis, we used Excel-2013® and SPSS v. 20.

## Results

Using our criteria of epidemics, it was possible to identify one wave CHIKV outbreaks in: Pernambuco (Jan-Apr 2016), Rio Grande do Norte (Feb-Jun 2016), and Noth-center Bahia (Feb-Mar 2016), Northeast Bahia (Jan-Mar 2016), North-Bahia (Sep 2015 - Apr 2016), South-Bahia (Jan-Apr 2016); and two waves outbreaks in: East-center Bahia (first Mar-Jul 2015, and second Feb-Mar 2016). Using the same criteria no epidemics was confirmed in other regions of Bahia (Table 1).

During CHIKV epidemics there was an increase in all-cause mortality in all analyzed regions, almost simultaneously with the peak of epidemics (Figure 1). Excess mortality rates were higher in the four region with the highest incidence rate of the chikungunya cases (table-1). In states of Pernambuco, South-Bahia, and Rio Grande do Norte incidence of dengue were high in 2015 and 2016. In Pernambuco, the highest incidence of dengue occurred in 2015, a year in which there were no excess deaths (Figure 1).

The excess mortality rates per 100,000 population and the excess deaths in the 4 critical regions studied were: Pernambuco (47.9 and excess death = 4,505) corresponding to the 21% increase in deaths, Rio Grande do Norte (42.5 and 1,478), South-Bahia (32.0 and 546), and North-Bahia (30.0 and 339) (Table 1). Table 2 shows excess mortality rates by age group for each region and Figure 2 presents time series incidence rate of chikungunya and mortality rate by age groups in Pernambuco.

**Figure:**
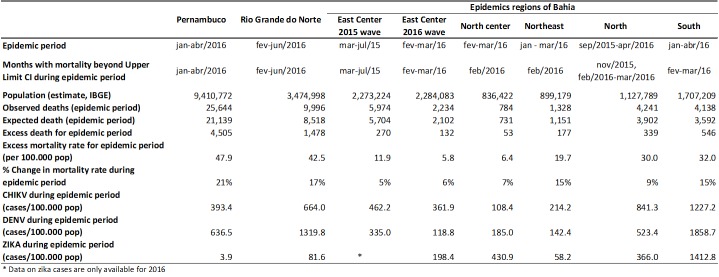
**Table 1:** Observed and expected deaths, all-causes mortality rates (deaths/100.000 population), excess mortality, change in mortality rate and incidence rate of chikungunya, zika, and dengue virus in 3 states of Northeast Brazil.

**Figure:**
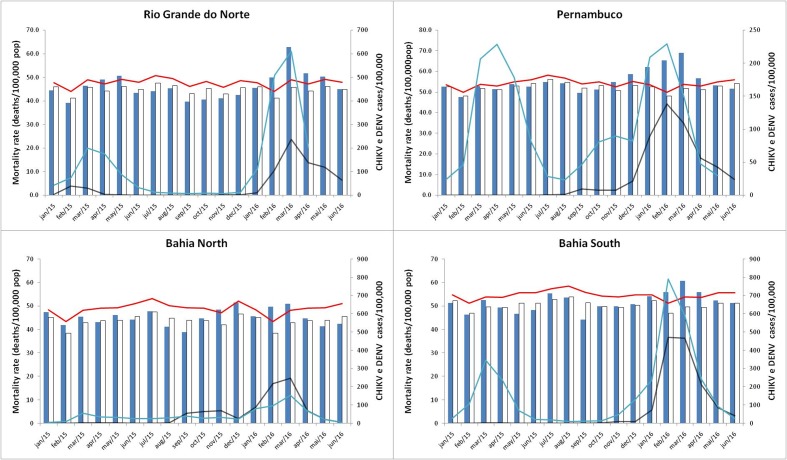
**Fig. 1:** Monthly observed Age Standardized Mortality Rate (ASMR, blue bar) and expected ASMR (white bar), 99% confidence interval (red line), monthly incidence rate of chikungunya (black line) and dengue (blue line) in Pernambuco, Rio Grande do Norte and Bahia (two regions).

**Figure:**
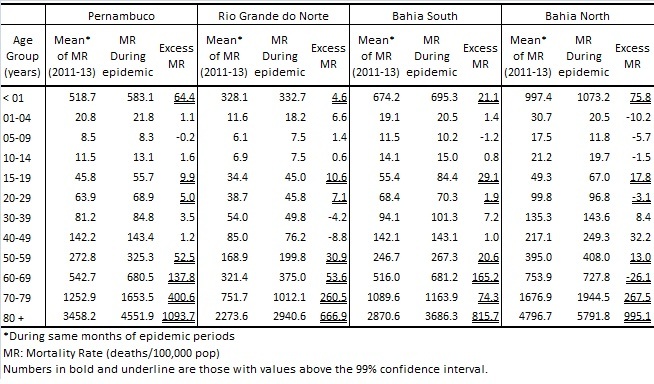
**Table 2:** Observed, expected all-causes mortality rates (deaths/100,000 population) and excess mortality by age group during epidemics periods in Pernambuco, Rio Grande do Norte and Bahia (two most affected regions, North and South).

**Figure:**
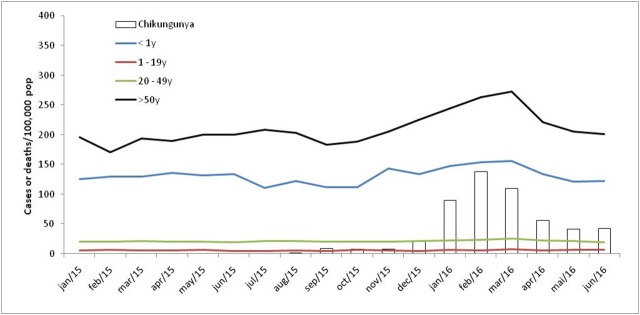
**Fig. 2:** Monthly all causes mortality rate by age group and monthly incidence rate of chikungunya from 2015 to 2016 in Pernambuco.

## Discussion

The understanding of the current chikungunya burden depends on a better understanding of the mortality associated with this arboviruses. Our findings show excess mortality during chikungunya epidemics in three most affected states in Brazil. The correlation between the incidence of chikungunya and increase in all-cause mortality reinforce the possibility that chikungunya virus has an impact on mortality rates. Similar results have already been found in other chikungunya epidemics in the past decades^2^^,^^3^^,^^4^. Incidence of dengue virus was high in all studied areas in the years 2011-2013, used as a baseline^5^. Therefore, we suggest that the excess deaths should be related to chikungunya infections and not another arbovirus that occurs at the same season. This is the first time that the excess mortality associated with chikungunya by age group has been calculated, and the results are very close to that found in the Reunion Islands epidemic using the data obtained in the death certificates^16^.

Since the chikungunya arrive in Americas, the virus has been identified in 44 countries and territories, leading to more than 2 million cases and 403 deaths^6^. In Brazil, the first case was confirmed in September 2014; however, the worst outbreak occurred in 2016, with 236,287 reported cases and 120 officially confirmed deaths^5^. The Brazilian Epidemiological Surveillance System detected far fewer deaths compared to our estimates in Pernambuco (54 deaths), Rio Grande do Norte (19 deaths), and all of Bahia (5 deaths)^5^. Mortality identified by the passive surveillance system was approximately 50 times lower than estimated from the excess of deaths. These differences may be due to failure to diagnose and difficulty recognizing severe forms of chikungunya^1^^,^^15^. Deaths caused by chikungunya occur more frequently in the elderly as a complication of preexisting diseases as reported in other hospital-based studies^7^^,^^8^. The present study showed that the increase in mortality was higher than the 99% confidence interval threshold in all regions in the age groups above 50 years. In fact the elderly are known to be the age group with the highest risk of severe forms and deaths due to chikungunya^7^. Because chikungunya is a disease not yet well known by health professionals in Brazil, the association of exacerbation of the underlying disease with chikungunya may have been underestimated leading to underreporting. In the age range between 15 and 19 years, there was an increase in mortality rate above the 99% confidence interval in 3 of the 4 regions studied. Clinical complications due to CHIKV infection have been reported in adults, not specifically in adolescents. Though, a systematic review on systemic infection due to CHIKV reports 52.2% and 37.1% articles respectively about cardiovascular and neurological manifestations^11^. On the other hand, severe cases and deaths of children and adolescents with postnatal zika virus infection are not commonly described in the literature^9^. However, Azevedo et al. (2016) draws attention to deaths in young people (16, 20 and 35 years old) with autoimmune diseases and immunodeficiency, besides the syndrome Guillain Barre^10^. Future investigations are necessary to understand better this occurrence.

There was also an increase, greater than 99% confidence interval threshold, in the mortality rate in children under 1 year. These latter findings had not been previously described and need to be better understood, however, it is not possible to exclude the possibility that the excess mortality in these age groups was related to the circulation of zika virus^12^.

The arbitrary definition of the epidemic period from 50 cases/100,000 could be less sensitive to express an epidemic context at lower incidence rates, however it was a parameter to enable comparisons between different periods and regions.

We must analyze results of time-series studies very carefully because of the method's own difficulties, which include time-coincident confounding factors. However, the periods of excess mortality observed do not coincide exactly with the seasons of influenza in the states of the Northeast region. Influenza AH1N1 was the most frequent viral subtype in 2016 and has not been found to be associated with high elderly mortality. Zika virus was not a reportable disease in 2015 there is no official data to calculate the IR of zika virus in that year, but even so the mortality associated with zika is admittedly low^13^. Other factors could be associated with excess mortality in that period; but no changes in the epidemiological profile of other diseases or events were reported in the studied regions. Furthermore, the overall arbovirus incidence may be underestimated due to sub diagnosis and under-reporting by health professionals. The impact of arbovirus co-circulation is still poorly understood^14^. The interaction of arboviruses, could theoretically result in more intense viremia or other immunological alterations.

Considering the magnitude and rapid territorial expansion of chikungunya transmission, we believe that global health systems should be prepared to manage the severity of the disease and associated deaths, not only acute and chronic joint disorders that have been the main concern related to chikungunya historically. Excess mortality is a useful indicator to quantify the impact of a health-related event and has traditionally been used to describe the increase of deaths during influenza season and extreme climates events^8^^,^^10^^,^^15^. All causes mortality during chikungunya epidemics could be monitored as a strategic tool, beyond individual case reporting to the epidemiological surveillance system, to assess excess mortality and the overall burden of chikungunya.

## Competing Interests

The authors have declared that no competing interests exist.

## Data Availability

Data are available from the figshare repository: https://doi.org/10.6084/m9.figshare.5397160.

## Corresponding Author

André Ricardo Ribas Freitas: arrfreitas2010@gmail.com

## References

[1] PAHO. 54th Directing Council Report on Chikungunya Virus Transmission and Its Impact in the Region of the Americas [Internet]. Washington, D.C., USA, 28 September-2 October 2015; 2015.

[2] Josseran L, Paquet C, Zehgnoun A, Caillere N, Tertre A Le, Solet J-L, et al. Chikungunya Disease Outbreak , Reunion Island. Emerg Infect Dis. 2006;12(12):1994–5. 10.3201/eid1212.060710 PMC3291364 17354339

[3] Mavalankar D, Shastri P, Bandyopadhyay T, Parmar J, Ramani K V. Increased Mortality Rate Associated with Chikungunya Epidemic, Ahmedabad, India. 2008;14(3). 10.3201/eid1403.070720 PMC2570824 18325255

[4] Beesoon S, Funkhouser E, Kotea N, Spielman A, Robich RM. Chikungunya Fever, Mautitius, 2006. Emerg Infect Dis. 2008;14(2):337–8. 10.3201/eid1402.071024 PMC2630048 18258136

[5] Ministério da Saúde do Brasil. Vigilância de A a Z [Internet]. Secretaria de Vigilância em Saúde. 2016 [cited 2016 Dec 22]. Available from: http://portalsaude.saude.gov.br/index.php/vigilancia-de-a-a-z

[6] Pan American Health Organization. PAHO WHO | Chikungunya | Statistic Data [Internet]. 2016 [cited 2016 Dec 13]. Available from: http://www.paho.org/hq/index.php?option=com_topics&view=readall&cid=5927&Itemid=40931&lang=en

[7] Economopoulou a, Dominguez M, Helynck B, Sissoko D, Wichmann O, Quenel P, et al. Atypical Chikungunya virus infections: clinical manifestations, mortality and risk factors for severe disease during the 2005-2006 outbreak on Réunion. Epidemiol Infect [Internet]. 2009 Apr [cited 2014 Jul 14];137(4):534–41. Available from: http://www.ncbi.nlm.nih.gov/pubmed/18694529 10.1017/S0950268808001167 18694529

[8] . Thompson WW, Shay DK, Weintraub E, Brammer L, Cox N, Anderson LJ. Mortality Associated With Influenza and Respiratory Syncytial Virus in the United States. JAMA. 2003;289(2):179–86. 10.1001/jama.289.2.179 12517228

[9] Ioos S, Mallet H-P, Leparc Goffart I, Gauthier V, Cardoso T, Herida M. Current Zika virus epidemiology and recent epidemics. Médecine Mal Infect. 2014 Jul;44(7):302–7. 10.1016/j.medmal.2014.04.008 25001879

[10] Pirard P, Vandentorren S, Pascal M, Laaidi K, Le Tertre A, Cassadou S, et al. Summary of the mortality impact assessment of the 2003 heat wave in France. Euro Surveill [Internet]. 2005 Jul [cited 2016 Dec 13];10(7):153–6. Available from: http://www.ncbi.nlm.nih.gov/pubmed/16088047 16088047

[11] Alvarez MF, Bolívar-Mejía A, Rodriguez-Morales AJ, Ramirez-Vallejo E. Cardiovascular involvement and manifestations of systemic Chikungunya virus infection: A systematic review." F1000Research 2017;6. 10.12688/f1000research.11078.1 PMC5405794 28503297

[12] Karwowski MP, Nelson JM, Staples JE, Fischer M, Katherine E. Fleming-Dutra E, et al. Zika virus disease: a CDC update for pediatric health care providers. Pediatrics 2016. Epub March 23, 2016. 10.1542/peds.2016-0621 27009036

[13] Pirard P, Vandentorren S, Pascal M, Laaidi K, Le Tertre A, Cassadou S, Ledrans M. Summary of the mortality impact assessment of the 2003 heat wave in France. Euro Surveill. 2005 Jul;10(7):153-6. PubMed PMID:16088047. 16088047

[14] Donalisio MR, Freitas ARR, Zuben APBV. Arboviruses emerging in Brazil: challenges for clinic and implications for public health. Revista de Saude publica, 2017; 51:1-6. 10.1590/S1518-8787.2017051006889 PMC5396504 28423140

[15] Cavalcanti LPG, Freitas ARR, Brasil P, Cunha RVD. Surveillance of deaths caused by arboviruses in Brazil: from dengue to chikungunya. Mem Inst Oswaldo Cruz. 2017 Aug;112(8):583-585. PubMed PMID:28767985. 28767985 10.1590/0074-02760160537 PMC5530552

[16] Renault P, Solet JL, Sissoko D, Balleydier E, Larrieu S, Filleul L, Lassalle C, Thiria J, Rachou E, de Valk H, Ilef D, Ledrans M, Quatresous I, Quenel P, Pierre V. A major epidemic of chikungunya virus infection on Reunion Island, France, 2005-2006. Am J Trop Med Hyg. 2007 Oct;77(4):727-31. PubMed PMID:17978079. 17978079

